# 15. Real-World Changes in *Clostridioides difficile* infection (CDI) Treatment Utilization and Clinical Outcomes Associated with Updated 2017 IDSA Guidelines among Medicare Beneficiaries in the U.S

**DOI:** 10.1093/ofid/ofab466.015

**Published:** 2021-12-04

**Authors:** Erik R Dubberke, Justin T Puckett, Engels N Obi, Sachin Kamal-Bahl, Kaushal Desai, Bruce Stuart, Jalpa A Doshi

**Affiliations:** 1 Washington University, St. Louis, Missouri; 2 COVIA Health Solutions, Lansdale, Pennsylvania; 3 Merck & Co., Rahway, New Jersey; 4 Merck & Co. Inc, Kenilworth, New Jersey; 5 University of Maryland, Baltimore, Baltimore, Maryland; 6 University of Pennsylvania, Philadelphia, Pennsylvania

## Abstract

**Background:**

The 2017 IDSA CDI guideline update phased out metronidazole (MTZ) and recommended vancomycin (VAN) or fidaxomicin (FDX) for first-line use. This study examined changes in CDI antibiotic use and clinical outcomes among Medicare beneficiaries with CDI pre- vs. post- the guideline update.

**Methods:**

This retrospective claims analysis used 2016-2018 national Medicare claims data. The two study samples included continuously eligible fee-for-service Medicare beneficiaries aged ≥66 years with a new CDI diagnosis followed by an antibiotic fill in the pre-period (04/01/2017-09/30/2017) and post-period (04/01/2018-09/30/2018), respectively. Outcomes included type of CDI antibiotic received; sustained response and CDI recurrence. Multivariable regressions compared pre- vs. post-period outcomes while controlling for sociodemographic and clinical factors.

**Results:**

The pre-period (N=7,389) and post-period (N=7,746) samples had similar characteristics (59% > 75 years, 32% male). Post-guideline update, absolute rates of MTZ use declined 27.7% (relative change [RC] -34.1%, p< 0.001) and VAN use increased 26.9% (RC +150.2%, p< 0.001) (Figure 1). While FDX use increased 0.8% (RC +87.8%, p< 0.001), overall use remained low (1.63%). Surprisingly, clinical outcomes did not improve between the pre- and post-period (Table 1). Even after adjustment, overall sustained response rates decreased (Odds Ratio [OR]: 0.93, p=0.0197) and overall CDI recurrence rates increased (OR: 1.13, p=0.0018) slightly in the post- vs. pre-period. Additional analyses by type of antibiotic showed that VAN (55.0% and 35.1%) was similar in outcomes to MTZ (54.2% and 33.0%), whereas FDX (71.4% and 20.9%) had higher sustained response and lower CDI recurrence rates, respectively (Figure 2).

Figure 1. First-line use of CDI treatments, pre- vs. post- the guideline update, among Medicare beneficiaries with CDI

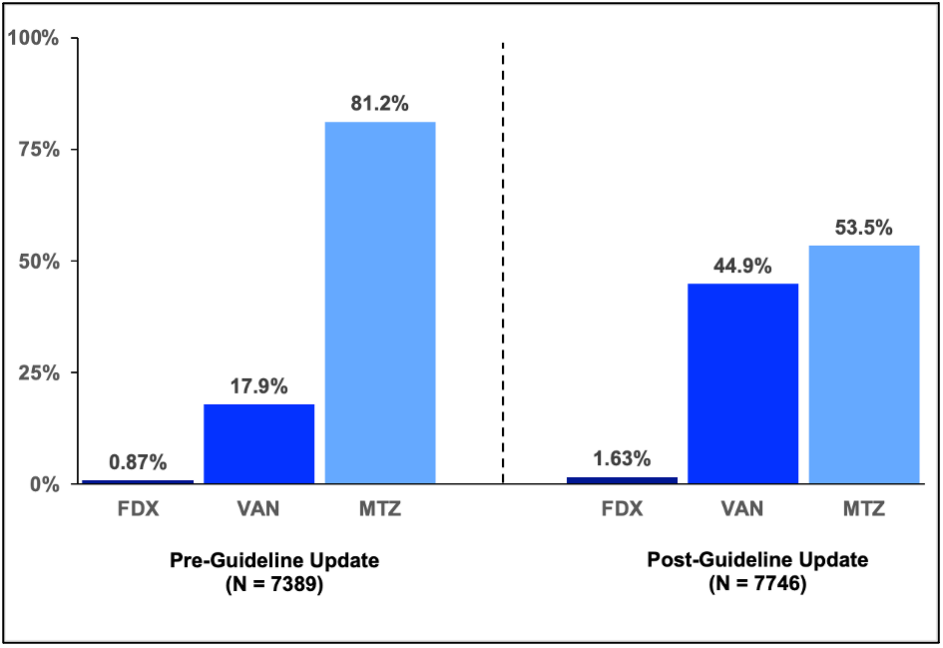

Table 1. Clinical outcomes, pre- vs. post- the guideline update, among Medicare beneficiaries with CDI

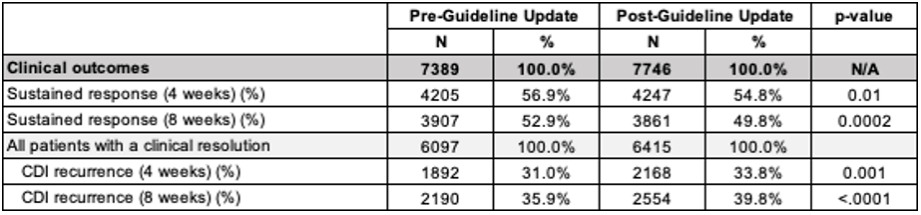

Figure 2. Clinical outcomes* by type of index CDI treatment among Medicare beneficiaries with CDI

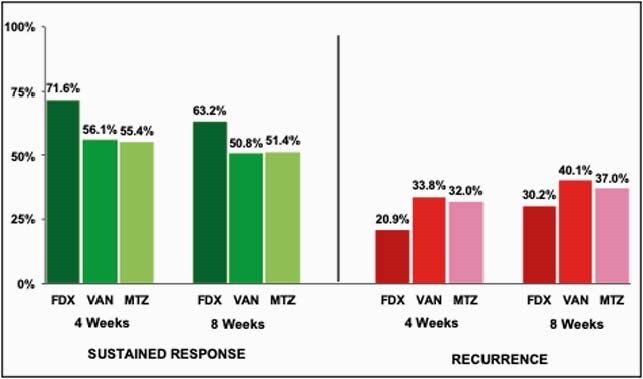

**Note:**

Pooled rates among patients on each index CDI treatment across the pre- and post-index periods.

**Conclusion:**

The 2017 IDSA guideline update was associated with a substantial increase in VAN use and decrease in MTZ use. FDX use rates remained low (< 2%). Overall CDI outcomes did not improve post guideline update despite the shift to guideline-indicated VAN. This may be because VAN was not associated with meaningfully improved outcomes relative to MTZ. However, improved outcomes seen with FDX relative to VAN and MTZ suggest potential benefits from its greater use in Medicare patients.

**Disclosures:**

**Erik R. Dubberke, MD, MSPH**, **Ferring** (Grant/Research Support)**Merck** (Consultant)**Pfizer** (Consultant, Grant/Research Support)**Seres** (Consultant)**Summit** (Consultant) **Justin T. Puckett, BA**, **COVIA Health Solutions** (Employee) **Engels N. Obi, PhD**, **Merck & Co.** (Employee, Shareholder) **Sachin Kamal-Bahl, PhD**, **AbbVie** (Consultant)**Arena Pharmaceuticals, Inc.** (Consultant)**COVIA Health Solutions** (Employee)**Janssen, Inc.** (Consultant)**Merck** (Consultant, Shareholder)**Novartis** (Consultant)**Pfizer, Inc.** (Consultant, Shareholder)**PhRMA** (Consultant) **Kaushal Desai, PhD**, **AstraZeneca Pharmaceuticals** (Shareholder)**Merck & Co. Inc.** (Employee) **Bruce Stuart, PhD**, **COVIA Health Solutions** (Consultant) **Jalpa A. Doshi, PhD**, **Acadia** (Consultant, Advisor or Review Panel member)**Allergan** (Advisor or Review Panel member)**Biogen** (Grant/Research Support)**Boehringer Ingelheim** (Other Financial or Material Support, Scientific lecture)**Catabasis** (Consultant)**Humana** (Grant/Research Support)**Janssen, Inc.** (Consultant, Grant/Research Support)**MeiraGTX** (Consultant)**Merck** (Grant/Research Support, Advisor or Review Panel member)**Novartis** (Grant/Research Support)**Otsuka** (Advisor or Review Panel member)**Regeneron** (Grant/Research Support)**SAGE Therapeutics** (Consultant)**Sanofi** (Grant/Research Support)**Shire** (Advisor or Review Panel member)**The Medicines Company** (Advisor or Review Panel member)

